# *Anaerococcus urinimassiliensis* sp. nov., a new bacterium isolated from human urine

**DOI:** 10.1038/s41598-021-82420-z

**Published:** 2021-01-29

**Authors:** Aurélie Morand, Mamadou Lamine Tall, Edmond Kuete Yimagou, Issa Isaac Ngom, Cheikh Ibrahima Lo, Florent Cornu, Michel Tsimaratos, Jean-Christophe Lagier, Anthony Levasseur, Didier Raoult, Pierre-Edouard Fournier

**Affiliations:** 1 Aix Marseille Université, IRD, AP-HM, MEФI, Institut Hospitalo-Universitaire Méditerranée-Infection, 19-21 Boulevard Jean Moulin, 13005 Marseille, France; 2 Pédiatrie Spécialisée Et Médecine Infantile, Hôpital de La Timone, AP-HM, Marseille, France; 3 Aix Marseille Université, IRD, AP-HM, SSA, VITROME, Institut Hospitalo-Universitaire Méditerranée-Infection, 19-21 Boulevard Jean Moulin, 13005 Marseille, France; 4 Pédiatrie Multidisciplinaire, Hôpital de La Timone, AP-HM, Marseille, France; 5 Institut Universitaire de France (IUF), Paris, France

**Keywords:** Microbiology, Medical research

## Abstract

To date there are thirteen species validly assigned to the genus *Anaerococcus*. Most of the species in this genus are anaerobic and of human origin. *Anaerococcus urinimassiliensis* sp. nov., strain Marseille-P2143^T^ is member of family *Peptoniphilaceae,* which was isolated from the urine of a 17-year-old boy affected by autoimmune hepatitis and membranoproliferative glomerulonephritis using the culturomic approach. In the current study, a taxono-genomics method was employed to describe this new species. The strain Marseille-P2143^T^ was gram positive cocci with translucent colonies on blood agar. Its genome was 2,189,509 bp long with a 33.5 mol% G + C content and exhibited 98.48% 16S rRNA similarity with *Anaerococcus provencensis* strain 9,402,080. When *Anaerococcus urinomassiliensis* strain Marseill-P2143^T^ is compared with closely related species, the values ranged from 71.23% with *A*. *hydrogenalis* strain DSM 7454^T^ (NZ_ABXA01000052.1) to 90.64% with *A. provencensis* strain 9402080^T^ (NZ_HG003688.1). This strain has implemented the repertoire of known bacteria of the human urinary tract.

## Introduction

The genus *Anaerococcus* belonging to the phylum *Firmicutes*, was first described in 2001^1^. Members of this bacterial genus are mainly anaerobic gram-positive cocci^2^. They are mostly encountered in human vagina, but can also be detected in nostrils or skin^3^. *Anaerococcus* spp. were involved in human infections and were isolated from different sites of human body such as peritoneal, ovarian and cervical abscesses, an arthritic knee, bacteremia, foot ulcers, a sternal wound and vaginoses^4–6^. Actually, the genus *Anaerococcus* contains 13 species validly described with standing in nomenclature^7^. The culturomic concept has recently been developed in our laboratory as an alternative method to expand the human gut repertoire through the multiplication of culture conditions with a rapid identification method by matrix-assisted laser desorption/ionization time-of-flight mass spectrometry (MALDI-TOF MS)^8–11^. Isolation and identification of microorganisms by culturomic can be used for further studies^12^ as well as for their diagnosis and/or therapeutic potential. The systematic description of new bacterial species recovered from patients may contribute to the description of emerging infections, but can also led to other discoveries. For example, the strain *Eubacterium limosum* isolated by culture in the gut sample has been used to test biotransformation’s of specific pollutants, methoxychlor and dichlorodiphenyltrichloroethane (DDT) insecticides^13^. The studies conducted on probiotic *Escherichia coli* strain Nissle 1917, show that a visceral analgesic can be produced by bacterial strain, which could be the basis of the development of new visceral pain therapies^14^. Currently clinical trials using bacterial cocktails are being used to restore dybiosis by fecal transplantation.

We report here, through a taxono-genomics strategy^15^, the description of *Anaerococcus urinimassiliensis* strain Marseille-P2143 (= CSUR P2143 = DSM 103,473), a new bacterium isolated from the urine of a young boy affected by autoimmune hepatitis associated with membranoproliferative glomerulonephritis, and classified into *Peptoniphilaceae* family. This new bacterial species was shortly described in a new species announcement in 2016^16^.

## Material and methods

### Sample collection and ethical considerations

In 2015, we isolated in the urine of a 17-year-old boy with autoimmune hepatitis and membranoproliferative glomerulonephritis, a bacterial strain belonging to the genus *Anaerococcus* that could not be identified at the species level. A signed and informed consent was collected from the patient and parents, and the study obtained approval from ethics committee of the Institut Fédératif de Recherche IFR48 under number 09-022. All the methods were carried out in accordance with relevant guidelines and regulations conformed to the Declaration of Helsinki.

### Strain isolation and identification by MALDI-TOF MS

**Figure 1:**
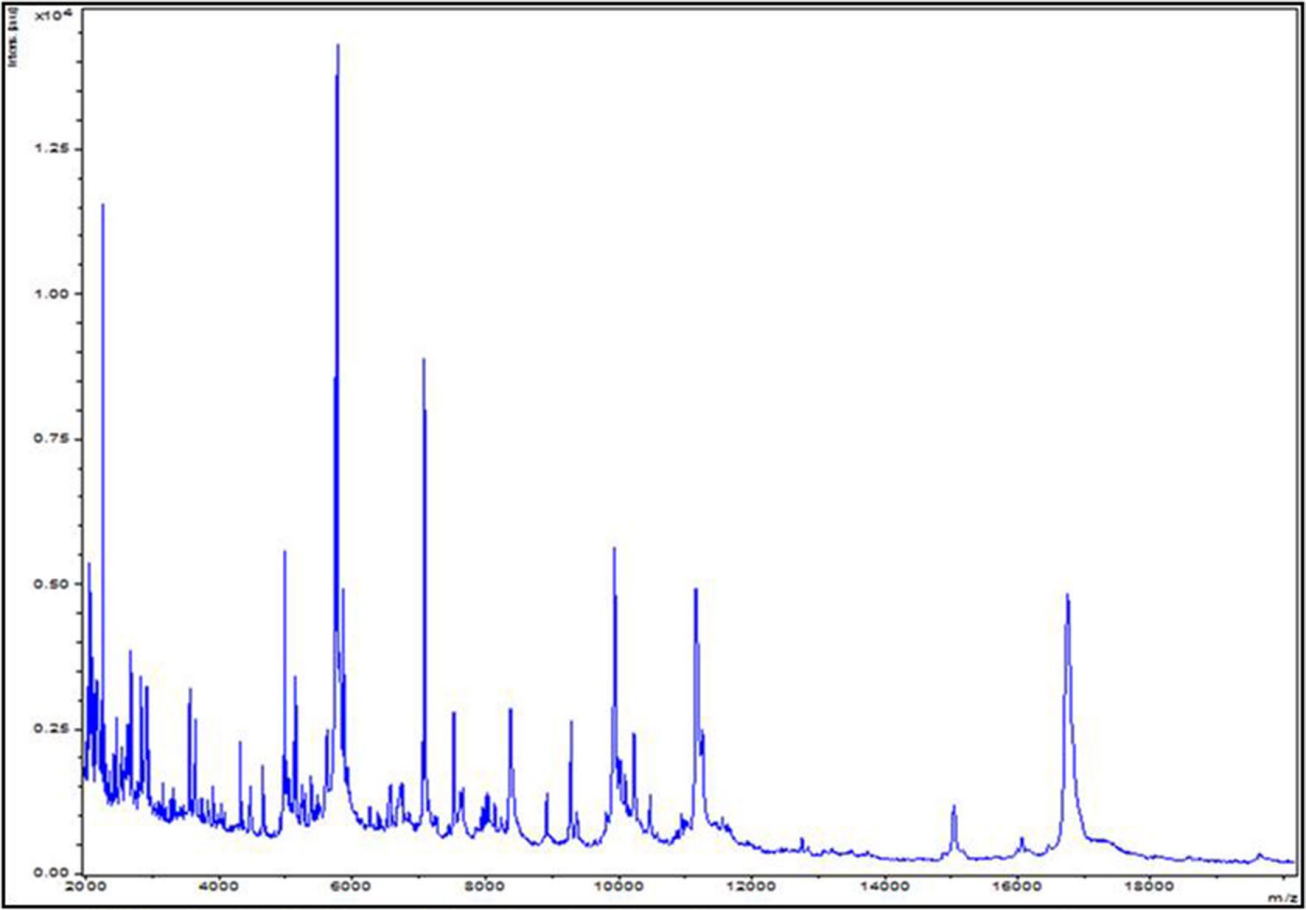
MALDI-TOF MS reference spectrum of *Anaerococcus urinimassiliensis* Marseille-P2143^T^, the reference spectrum was generated by comparison of spectra from 12 individual colonies using the Biotyper 3.0 software.

The initial growth was obtained after 10 days of incubation in an anaerobic blood culture vial (Becton Dickinson, Le Pont-de-Claix, France) supplemented with 5 mL of 0.2 μm filtered rumen fluid. A pure culture of strain Marseille-P2143 was then obtained after 48 h of incubation at 37 °C on 5% sheep blood–Columbia agar medium (bioMérieux, Marcy l'Etoile, France) in anaerobic atmosphere generated using the GENbag Anaer system (bioMérieux) as previously described^16^. Strain Marseille-P2143^T^ was not identified by Matrix Assisted Laser Desorption Ionization-Time of Flight Mass Spectrometry (MALDI-TOF MS), after several attempts essayed as described elsewhere^17^. The screening was performed on a Microflex LT spectrometer (Bruker, Daltonics, Bremen, Germany) as previously reported^18^. The reference spectrum obtained (Fig. 1) was imported and analyzed using the Biotyper software (version 3.0)^19^ against the Bruker database, which was continually incremented with local URMS database (https://www.mediterranee-infection.com/urms-data-base/).

### Strain identification and phylogenetic tree

In order to classify this bacterium, the 16S rRNA gene was amplified using the primer pair fD1 and rP2 (Eurogentec, Angers, France) and sequenced using the Big Dye Terminator v1.1 Cycle Sequencing Kit and 3500xLGenetic Analyzer capillary sequencer (Thermofisher, Saint-Aubin, France) as previously described^12^. The 16S rRNA nucleotide sequence was assembled and corrected using CodonCode Aligner software (http://www.codoncode.com). For phylogenetic analysis, sequences of the phylogenetically closest species were obtained after performing a BLASTn search within the NCBI Blastn 16S rRNA Sequence Reference Base for closest related species to calculate sequence similarities of the 16S rRNA genes (refseq_rna) (https://blast.ncbi.nlm.nih.gov/Blast.cgi?PROGRAM=blastn&PAGE_TYPE=BlastSearch&LINK_LOC=blasthome). “The All‐Species Living Tree" Project of Silva^20^. The alignment was performed using MUSCLE^21^. The evolutionary history was inferred using the Maximum Likelihood method based on the Tamura-Nei model^22^. The tree with the highest log likelihood (-5398.79) is shown. The percentage of trees in which the associated taxa clustered together is shown next to the branches. Initial tree(s) for the heuristic search were automatically obtained by applying Neighbor-Join and BioNJ algorithms to a matrix of pairwise distances estimated using the Maximum Composite Likelihood (MCL) approach, and then selecting the topology with superior log likelihood value. The tree is drawn to scale, with branch lengths measured in the number of substitutions per site. The analysis involved 18 nucleotide sequences. The codon positions included were 1st + 2nd + 3rd + Noncoding. All positions containing gaps and missing data were eliminated. There were a total of 1184 positions in the final dataset. Evolutionary analyses were conducted in MEGA software (version X) (https://www.megasoftware.net/)23.

### Phenotypic characteristics and biochemical features

The optimum growth condition of the strain was determined by culturing the strain under different temperatures, atmospheres, PH and salinity. The strains were cultured and incubated under aerobic, anaerobic (GENbag anaer, bioMérieux Limited, France) and microaerophilic (GENbag Microaer, bioMérieux Limited, France) conditions on Columbia agar enriched with 5% sheep blood (bioMérieux Limited, France) at the following temperatures: 25, 28, 37, 45, and 55 °C. The pH conditions used were 5.5, 6, 6.5, 7, 7.5, 8, 8.5, 9, 9.5 and the salinity conditions used were the following: 0%, 5%,10%, 25%, 50%, 100%. The phenotypic characteristics of the strain such as Gram staining, motility, oxidase and catalase activities were determined using standard microbiological methods as previously described^23^. These phenotypic and biochemical characteristics were tested for strain Marseille-P2143 incubated at 37 °C for 48 h. The use of carbon sources was assayed with API 50 CH strips. The API 50 CH strips were interpreted after incubation at 37 °C for 24 h. Antibiotic susceptibility was determined using disc diffusion plate method according to the instructions of the CA-SFM / EUCAST 2020 (https://www.eucast.org/clinical_breakpoints_and_dosing/eucast_setting_breakpoints/), in reference to the EUCAST disk diffusion method for susceptibility testing of the Bacteroides fragilis group isolates^24^. Antibiotic discs used were the following: erythromycin (15 μg/ml), penicillin G (10 UI), doxycycline (30 μg/ml), rifampicin (30 μg/ml), vancomycin (30 μg/ml), clindamycin (15 μg/ml), fosfomycin (50 μg/ml), amoxicillin (25 μg/ml), colistin (15 μg/ml), gentamycin (500 μg/ml), amoxicillin-clavulanic acid (30 μg/ml), ceftriaxone (30 μg/ml), colistin (50 μg/ml), trimethoprim-sulfamethoxazole (25 μg/ml), oxacillin (5 μg/ml), imipenem (10 μg/ml), tobramycin (10 μg/ml), and metronidazole (4 μg/ml). For fatty acids analysis, the bacterial strains cultivated on cos medium after 48 h in aerobic condition, were collected in triplicates in three tubes with approximately the same amount of biomass and were then weighed. We obtained an average of 130 mg of biomass per tube and cellular fatty acid methyl ester (FAME) analysis was performed by Gas Chromatography/Mass Spectrometry (GC/MS) as described by Sasser et al.^25^. GC/MS analyses were carried out as previously described^26^. Spectral database search was performed using MS Search 2.0 operated with the Standard Reference Database 1A (NIST, Gaithersburg, USA) and the FAMEs mass spectral database (Wiley, Chichester, UK).

### Genome sequencing and assembly

Genomic DNA was extracted using the EZ1 biorobot with the EZ1 DNA tissue kit (Qiagen, Hilden, Germany) and then sequenced on a MiSeq sequencer (Illumina Inc, San Diego, CA, USA) with the Nextera Mate Pair sample prep kit and Nextera XT Paired End (Illumina), as previously described^27^. The assembly was performed using a pipeline containing several softwares (Velvet^28^, SPAdes^29^ and SOAP Denovo^30^) and trimmed (MiSeq and Trimmomatic^31^ softwares) or untrimmed data (only MiSeq software). GapCloser software^32^ was used to reduce assembly gaps. Scaffolds < 800 base pairs (bp) and scaffolds with a depth value lower than 25% of the mean depth were removed. The best assembly was selected using different criteria (number of scaffolds, N50, number of N). The degree of genomic similarity of strain Marseille-P2143^T^ (NZ_FQRX00000000.1) with closely related species *Anaerococcus hydrogenalis DSM 7454*^*T*^*(NZ_ABXA01000052.1)*, *Anaerococcus jeddahensis strain SB3*^*T*^*(NZ_CWHU01000001.1)*, *Anaerococcus tetradius ATCC 35,098T (NZ_GG666394.1)*, *Anaerococcus octavius strain NCTC9810*^*T*^*(UFTA01000001.1), Anaerococcus pacaensis 9403502*^*T*^*(NZ_HG326663.1)*, *Anaerococcus prevotii strain NCTC11806*^*T*^* (UFSY01000001.1)*, *Anaerococcus provencensis strain 9402080*^*T*^*(NZ_HG003688.1), Anaerococcus rubiinfantis MT16*^*T*^*(FAVH01000001.1)*, *Anaerococcus senegalensis JC48*^*T*^* (NZ_HE578907.1)*, was estimated using the OrthoANI software^33^. These genomes were then aligned with scapper (https://github.com/tseemann/scapper). These aligned genomes were eventually used to build the phylogenetic tree using the Maximum likelihood method from MEGA X software^34^.

### Genome annotation and analysis

The prediction was performed using prodigal in the open reading frame (ORF)^35^ with default parameters. Planned ORFs covering a sequencing gap region (containing N) were excluded. The bacterial proteome was predicted with BLASTP (E-value of 1e03, coverage of 0.7 and identity percentage of 30) against the database of orthologic group clusters (COG). If no matches were found, we searched the nr database^36^ using BLASTP with an E-value of 1e03, a coverage of 0.7 and an identity percentage of 30. An E-value of 1e05 was used only if the sequence length was less than 80 amino acids (aa). The domains maintained by the PFAM (PFAM-A and PFAM-B domains) were searched on each protein using the hmmscan analysis tool. RNAmmer^37^ and the tRNAScanSE tools^38^ were used to find rRNA and tRNA genes. When BLASTP E-value was lower than 1e-03 for alignment length greater than 80 amino acids, ORFans are identified. However, when alignment lengths smaller than 80 amino acids were obtained, an E-value of 1e-05 was used. Artemis^39^ was used for data management and visualization of genomic characteristics. The in-house MAGI software was used to analyze the average level of similarity of nucleotide sequences at the genome level. It calculated the average genomic identity of gene sequences (AGIOS) among the genomes compared^15^. This software combines Proteinortho software^40^ to detect orthologic proteins in pairwise genomic comparisons. Then, the corresponding genes were recovered and the average percentage identity of nucleotide sequences among the orthological ORFs was determined using the Needleman—Wunsch global alignment algorithm. We also used the Genome-to-Genome Distance Calculator Web service to calculate DNA: Digital DNA hybridization estimates (dDDH) with confidence intervals according to recommended parameters (Formula 2, BLAST)^41^.

The degree of genomic similarity of Marseille-P2143 with closely related species was estimated using the OrthoANI software^42^.

### Ethical approval

All the methods were carried out in accordance with relevant guidelines and regulations conformed to Declaration of Helsinki.

## Results and discussion

### Identification of Strain Marseille-P2143

**Figure 2:**
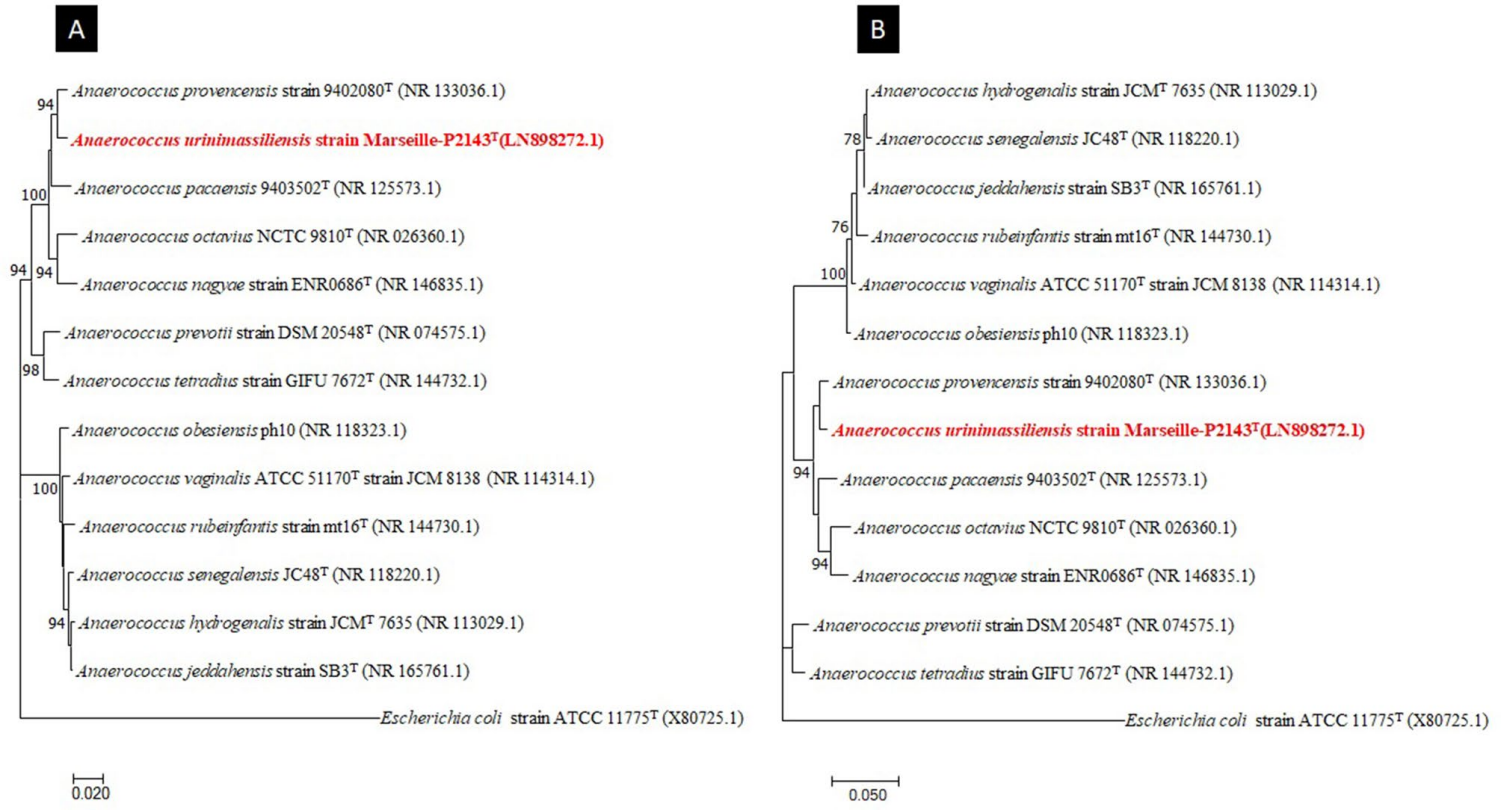
Phylogenetic tree highlighting the position of *Anaerococcus urinimassiliensis* Marseille-P2143^T^ sp. nov., with regard to other closely related species. Genbank accession numbers of 16S rRNA are indicated in parentheses. Sequences were aligned using MUSCLE with default parameters, phylogenetic inference were obtained using the neighbor joining (**A**) and Maximum likelihood (**B**) method and the MEGA X software. Bootstrap values obtained by repeating the analysis 50 times to generate a majority consensus tree are indicated at the nodes. The scale bar indicates a 2% and 5% nucleotide sequence divergence.

The mass spectrum of strain Marseille-P2143 was not present in the MALDI-TOF MS Bruker database. Thus, we were not able to identify this strain using this instrument. However, the 16S rRNA gene sequencing analysis indicated that it exhibited 98.48% sequence similarity with Anaerococcus provencensis strain 9402080T (Genbank accession number NR_133036.1), the phylogenetically closest species after blast in the NCBI database (Fig. 2). We consequently proposed to classify strain Marseille-P2143 Type strain as a new species within the genus *Anaerococcus*^43^.

### Phenotypic characteristics and biochemical features

**Figure 3:**
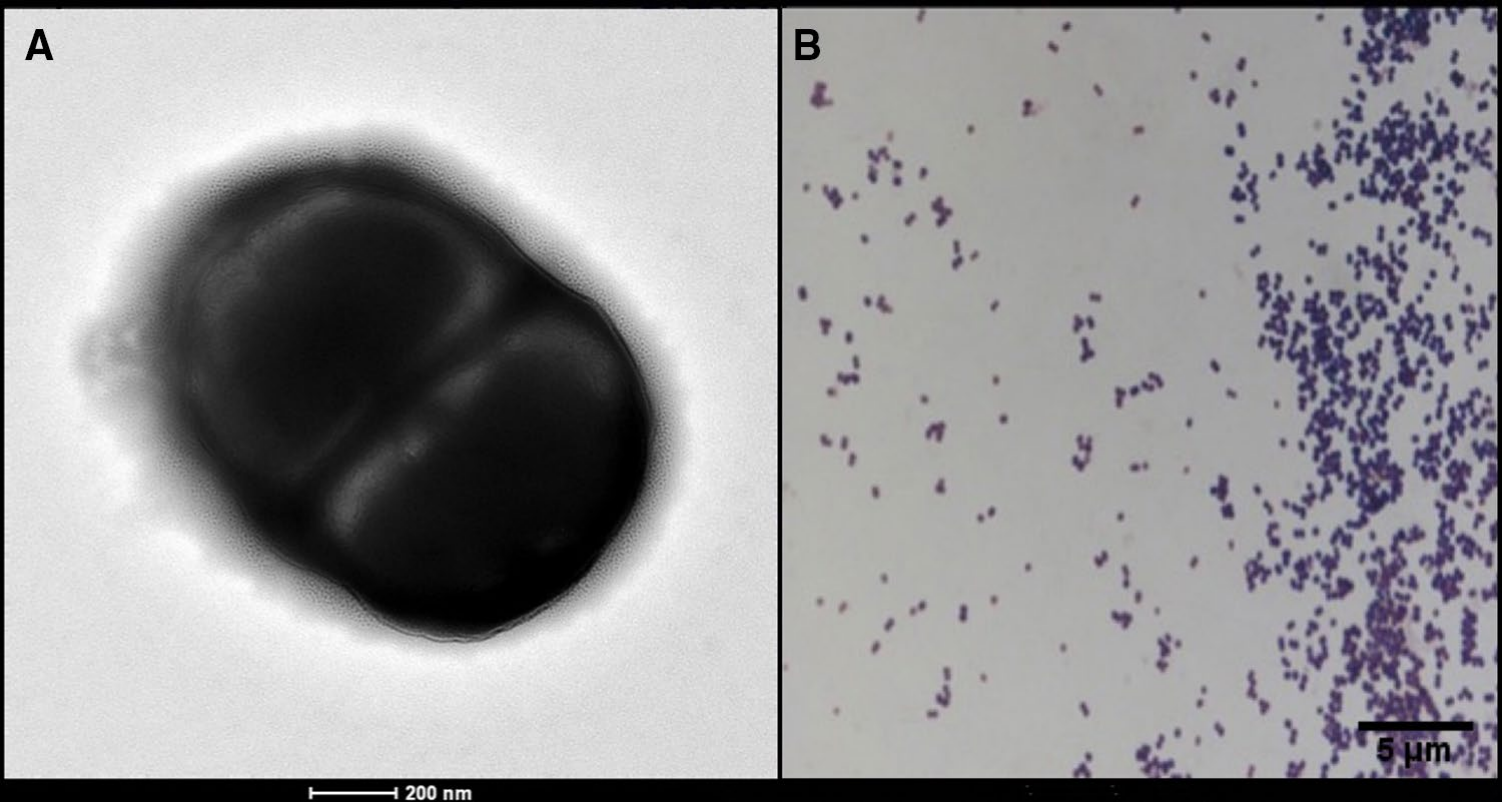
Morphology of *Anaerococcus urinimassiliensis* sp.nov. (**A**) Scanning electron microscopy of stained *Anaerococcus urinimassiliensis* sp. nov., Marseille-P2143^T^. (**B**) Gram staining of *Anaerococcus urinimassiliensis* sp. nov., Marseille‐P2143^T^.

The optimal growth of strain Marseille-P2143 was obtained after 5 day of culture at 37 °C under anaerobic conditions (anaeroGEN, Oxoid Ltd, Dardilly, France). Agar-grown colonies were small with a mean diameter of 50 μm and were translucent white. Bacterial cells were Gram-positive cocci ranging in diameter from 0.5 to 0.7 µm (Fig. 3). Catalase and oxidase activities were not observed. A brief description and characteristics of strain Marseille-P2143 are summarized in Table 1. Using API ZYM strip, positive reactions were observed for alkaline phosphatase, esterase, esterase lipase, leucine arylamidase, cystine arylamidase, trypsin, naphthol-AS-BI-phosphohydrolase, α-galactosidase and β-galactosidase. But negative reactions were noted with lipase, valine arylamidase, α-chymotrypsin, acid phosphatase, and β-glucuronidase. Using API 50 CH strip (bioMérieux), strain Marseille-P2143 was able to metabolize d-galactose, d-glucose, d-fructose, *N*-acetyl-glucosamine, salicin, d-maltose, d-lactose, and d-saccharose. Negative reactions were obtained for glycerol, d-mannose, methyl α-d-glucopyranoside, d-turanose, d-tagatose, erythritol, d-adonitol, d-mannose, mannitol, d-sorbitol, amygdalin, arbutin, esculin, d-melibiose, d-trehalose, inulin, d-melezitose, d-raffinose, starch, glycogen, d-arabitol, l-arabitol and Potassium 5-ketogluconate. When strain Marseille-P2143 was compared to *Anaerococcus provencensis* strain 9402080^T^ the closest species with a validly published name, it differed in catalase activity (Table 2). The Antimicrobial susceptibility test according to the EUCAST showed that strain Marseille-P2143 was susceptible to Penicillin, Oxacillin, Amoxicillin, Amoxicillin-Clavulanic acid, Imipenem, Rifampin, Vancomycin, Clindamycin, Fosfomycin, and Tobramycin but resistant to Ceftriaxone, Erythromycin, Doxycycline, Gentamicin, Colistin and Trimethoprim-Sulfamethoxazole. Hexadecanoic acid was the most abundant fatty acid (57%). Unsaturated fatty acids 9-Octadecenoic acid and 9,12-Octadecadienoic were also detected with significant amounts. Tetradecanoic acid and Octadecanoic acid were minor fatty acids were quantified. By comparing strain Marseille-P2143 with related close species (Table 3), we obtain mainly a similar profile between species with C_16:0_ as the abundant fatty acid (> 50%).

**Table 1:** Description and characteristics of *Anaerococcus urinimassiliensis* Marseille-P2143^T^.

Property	Terms
Genus name	*Anaerococcus*
Species name	*Anaerococcus urinimassiliensis*
Specific epithet	*Urinimassiliensis*
Species status	sp. nov
Designation of the type strain	Marseille-P2143^T^
Strain collection numbers	CSUR P2143, DSM 103,473
16S rRNA gene accession number	LN898272
Genome accession number	FQRX01000001
Genome size	2,190,108 bp
G + C (mol %)	33.47
Origin	Marseille, France
Date of isolation	2015
Source of isolation	Human urine sample
Conditions used for standard cultivation	Columbia agar + with 5% sheep blood for 48 h of incubation
Gram stain	Positive
Cell shape	Cocci
Cell size (diameter)	0.5–0.7 (μm)
Motility	Nonmotile
Colony morphology	Transluscent white
Temperature optimum	37 °C
pH range	5.5–8
Relationship to O_2_	Anaerobe
Oxidase	Negative
Catalase	Negative
Salinity range	< 5%

**Table 2:** Differential phenotypic characteristics of *A. urinimassiliensis* sp. nov, Marseille-P2143^T^ and related species of the genus *Anaerococcus*.

Characteristics	1	2	3	4
Cell diameter (µm)	0.5–0.7	0.9–1.3	0.6–1.5	0.9–1.4
Oxygen requirement	Anaerobic	Anaerobic	Anaerobic	Anaerobic
Gram stain	+	+	+	+
Motility	−	−	−	−
Production of:				
Alkaline phosphatase	+	+	−	+
β-galactosidase	+	+	−	na
Indole	−	−	−	−
Catalase	−	+	+	+
Acid form:				
Mannose	−	−	+	−
Glucose	+	+	+ /−	−
Lactose	+	+	−	−
Raffinose	−	−	+	−
G + C content (mol%)	33.47	33.48	33	35.05
Origin	Human urine	Human cervical abscess	Human plasma	Human blood

**1**, *A. urinimassiliensis* sp. nov, Marseille-P2143^T^; **2**, *A. provencensis* 9402080^T^; **3**, *A. prevotii* DSM 20548^T^; **4**, *A. pacaensis* 9403502^T^.

+ : positive reaction; -: negative reaction; na: not available.

**Table 3:** Cellular fatty acid composition (%) of *Anaerococcus urinimassiliensis* sp. nov, Marseille-P2143^T^ compared with other *Anaerococcus* species.

Fatty acids	Name	*A. urinimassiliensis*	*A. jeddahensis*	*A. rubiinfantis*	*A. vaginalis*	*A. senegalensis*
16:00	Hexadecanoic acid	56.8	51.7	52.7	59.2	54.6
18:1n9	9-Octadecenoic acid	13.6	13.6	28.2	18.8	17.6
18:2n6	9,12-Octadecadienoic acid	13.5	16.4	10.1	6.5	9.2
14:00	Tetradecanoic acid	5.4	7.7	2.1	6.9	4.4
18:00	Octadecanoic acid	4.7	2.6	3.6	5.7	11.5
18:1n7	11-Octadecenoic acid	2.4	1.5	ND	ND	ND
15:00	Pentadecanoic acid	TR	1.2	TR	1.0	TR
16:1n7	9-Hexadecenoic acid	TR	TR	1.1	TR	ND
12:00	Dodecanoic acid	TR	1.1	TR	TR	TR
10:00	Decanoic acid	TR	2.8	ND	1.2	TR

*TR* trace amounts < 1%, *ND* not detected.

### Genome properties

**Figure 4:**
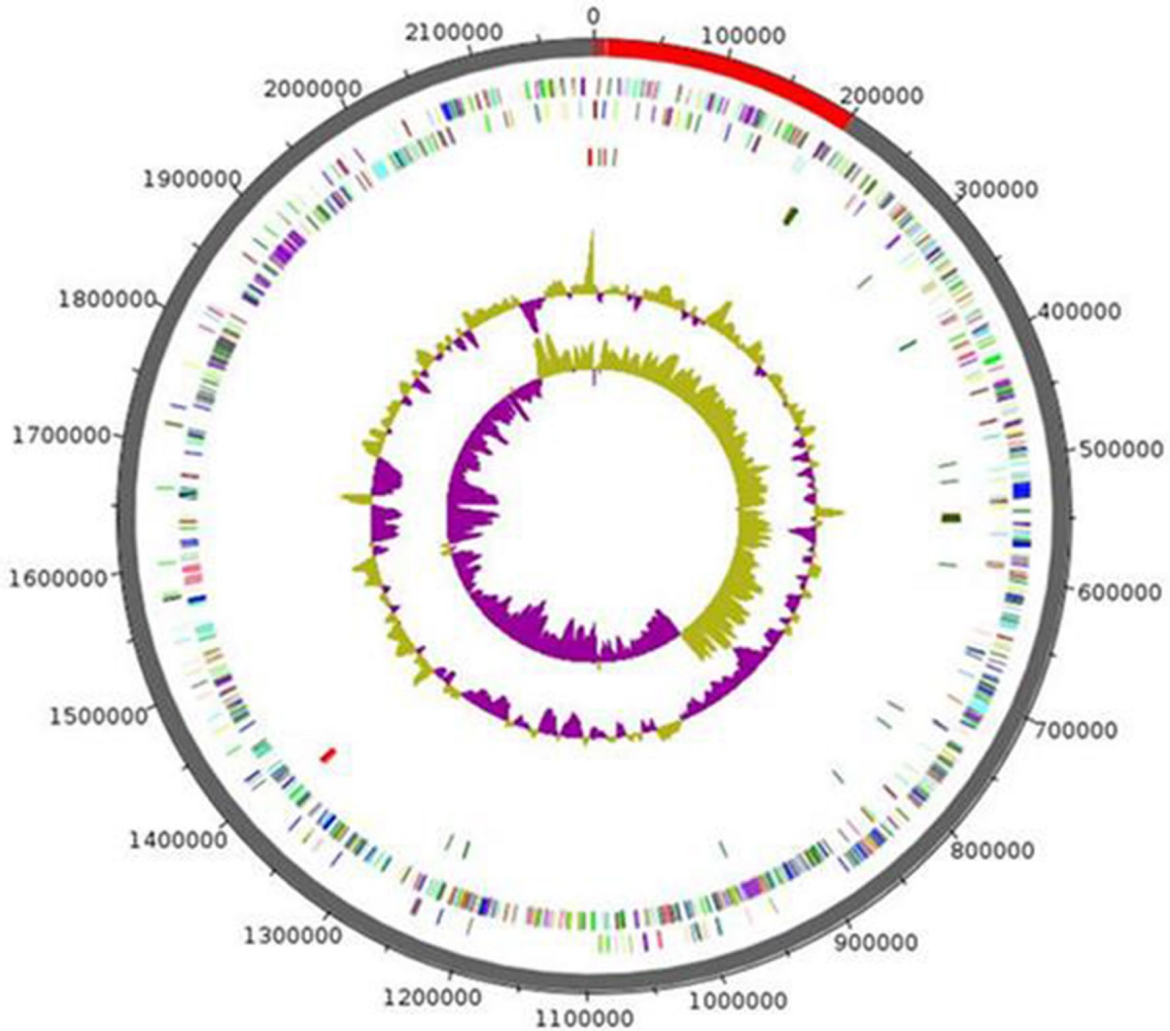
Graphical circular map of the chromosome of *Anaerococcus urinimassiliensis* sp.nov. From outside to the center: genes on the forward strand colored by COG categories (only genes assigned to COG), genes on the reverse strand colored by COG categories (only gene assigned to COG), RNA genes (tRNAs green, rRNAs red), GC content and GC skew.

The genome of Marseille-P2143 was 2,189,509 bp long with 33.5 mol% G + C content (Fig. 4). It was composed of 10 scaffolds (11 contigs). Of the 2077 predicted genes, 1952 were protein-coding genes and 61 were RNAs (4 genes are 5S rRNA, 3 genes are 16S rRNA, 5 genes are 23S rRNA, 46 genes are tRNA genes) 64 were pseudo genes.

### Genome comparison

**Figure 5:**
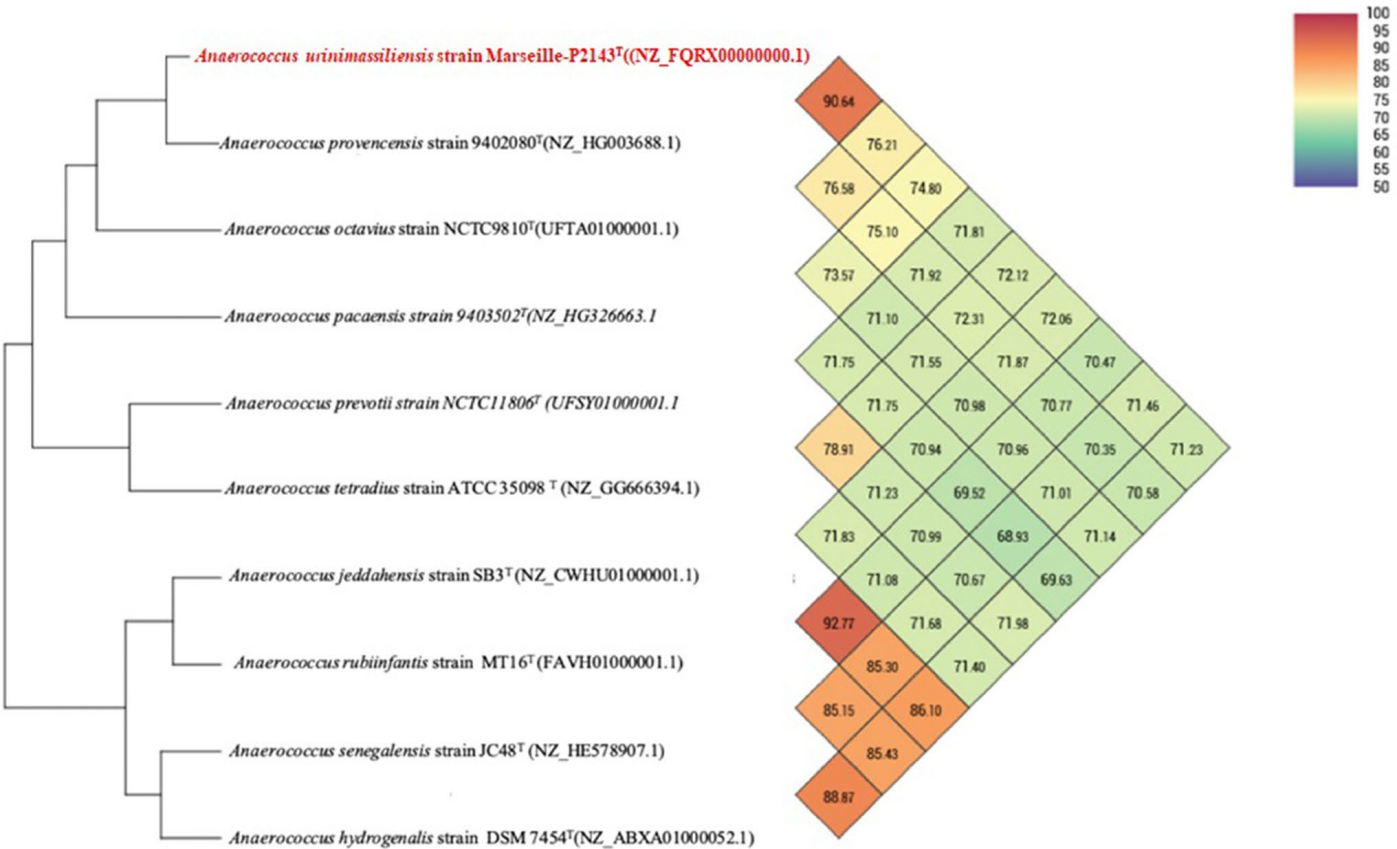
Heatmap generated with OrthoANI values calculated using the OAT software between *Anaerococcus urinimassiliensis* Marseille-P2143^T^ sp. nov. and other closely related species with standing in nomenclature.

**Figure 6:**
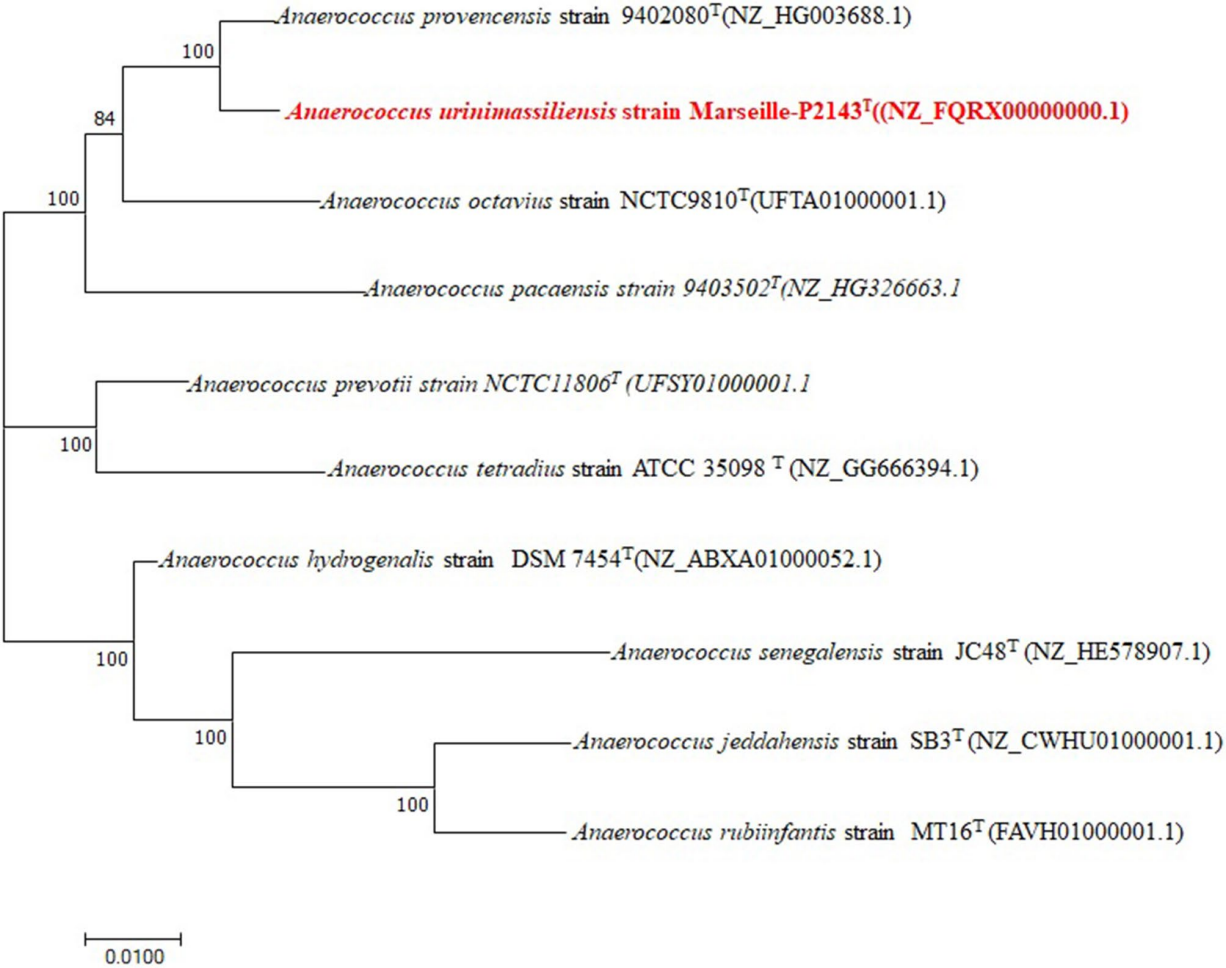
Phylogenetic tree highlighting the position of *Anaerococcus urinimassiliensis* Marseille-P2143^T^ sp. nov., with regard to other closely related species considering the genome. Genbank accession numbers of genome are indicated in parentheses. Sequences were aligned using scraper with default parameters, phylogenetic inference were obtained using the Maximum likelihood method and the MEGA X software. Bootstrap values obtained by repeating the analysis 50 times to generate a majority consensus tree are indicated at the nodes. The scale bar indicates a 1% nucleotide sequence divergence.

The draft genome sequence of *Anaerococcus urinimassiliensis* (2,189,509 bp) was larger than that of *A. prevotii* strain NCTC11806^T^ (UFSY01000001.1) (2,004,977 bp), *A. pacaensis* strain 9403502^T^(NZ_HG326663.1) (845,487 bp) and *A. provencensis* strain 9402080^T^(NZ_HG003688.1) (29,418). The G + C content of *A. urinimassiliensis* strain Marseille-P2143^T^ (33.5 mol%) was smaller than those of *A. provencensis* strain 9402080^T^(NZ_HG003688.1) (29,418) (33.7%), *A.pacaensis* strain 9403502^T^(NZ_HG326663.1) (34.1%) and *A. prevotii* strain NCTC11806^T^ (UFSY01000001.1) (35.6%). The gene content of *A. urinimassiliensis* strain Marseille-P2143 (2077genes) was smaller than those of *A. provencensis* strain 9402080^T^(NZ_HG003688.1) (2146) and *A.pacaensis* strain 9403502^T^(NZ_HG326663.1) (2223), but larger than those of *A. prevotii* strain NCTC11806^T^ (UFSY01000001.1) (1894) respectively (Table 4). OrthoANI values among closely related species (Figs. 5) ranged from 69.52% between *A. pacaensis* strain 9403502^T^(NZ_HG326663.1) and *A. rubiinfantis* strain MT16^T^ (FAVH01000001.1) to 92.77% between *A. jeddahensis* strain SB3^T^ (NZ_CWHU01000001.1) and *A. rubiinfantis* strain MT16^T^ (FAVH01000001.1). When *Anaerococcus urinomassiliensis* strain Marseill-P2143is compared with closely species, the values ranged from 71.23% with *A*. *hydrogenalis* strain DSM 7454^T^ (NZ_ABXA01000052.1) to 90.64% with *A. provencensis* strain 9402080^T^ (NZ_HG003688.1). The phylogenetic tree highlighting the position of the genome of strain Marseille-P2143 in relation to other closely related species with a validly published name is presented in (Fig. 6). Genes with putative function (by COGs) were 1796 for *Anaerococcus urinimassiliensis* strain Marseille-P2143^T^ (81%), ,903 for *A.provencensis* strain 9402080^T^(NZ_HG003688.1) (82%), 1539 for *A.prevotii* strain NCTC11806^T^ (UFSY01000001.1) (86) and 1867 for *Anaerococcus pacaensis* strain 9403502^T^(NZ_HG326663.1) (78%). Finally, 414, 426, 253 and 538 genes (19%,18%, 14% and 22%) were annotated as hypothetical proteins for *Anaerococcus urinimassiliensis* strain Marseille-P2143, *A.provencensis* strain 9402080^T^(NZ_HG003688.1), *A.prevotii* strain NCTC11806^T^ (UFSY01000001.1) and *A.pacaensis* strain 9403502^T^(NZ_HG326663.1), respectively Table 5. Analysis of the Clusters of Orthologous Groups (COGs) categories shows that the mobile elements of the Marseille-P2143 genome appear to be more numerous than those of the genomes of *A.provencensis* strain 9402080^T^(NZ_HG003688.1), *A.prevotii* strain NCTC11806^T^ (UFSY01000001.1), *A.pacaensis* strain 9403502^T^(NZ_HG326663.1) (82, 45, 16 and 48 in category [X], respectively).

**Table 4:** Comparison of genome size, G + C mol% content and gene count of Marseille-P2143^T^ with other species within the family *Peptoniphilaceae*.

Species	Size (bp)	G + C (%mol)	Total of genes
*Anaerococcus provencensis* strain 9402080^T^	29,418	33.7	2146
*Anaerococcus prevotii* strain NCTC11806^T^	2,004,977	35.6	1894
*Anaerococcus pacaensis* strain 9403502^T^	845,487	34.14	2223
*Anaerococcus urinimassiliensis strain Marseille-P2143*	2,189,509	33.5	2077

**Table 5:** Number of genes associated with the 25 general clusters of orthologous group (COG) functional categories of strain Marseille-P2143 with other species within the family *Peptoniphilaceae*.

*Code*	*Anaerococcus urinimassiliensis*	*Anaerococcus provencensis*	*Anaerococcus prevotii*	*Anaerococcus pacaensis*	Description
*J*	188	184	176	191	Translation, ribosomal structure and biogenesis
*A*	0	0	0	0	RNA processing and modification
*K*	128	152	115	142	Transcription
*L*	112	107	98	110	Replication, recombination and repair
*B*	1	1	1	1	Chromatin structure and dynamics
*D*	30	31	30	31	Cell cycle control, cell division, chromosome partitioning
*Y]*	0	0	0	0	Nuclear structure
*V*	93	96	72	111	Defense mechanisms
*T*	77	100	70	74	Signal transduction mechanisms
*M*	90	88	71	72	Cell wall/membrane/envelope biogenesis
*N*	8	11	7	10	Cell motility
*Z*	1	1	1	2	Cytoskeleton
*W*	3	5	4	4	Extracellular structures
*U*	21	23	15	21	Intracellular trafficking, secretion, and vesicular transport
*O*	76	75	71	83	Posttranslational modification, protein turnover, chaperones
*X*	82	45	16	48	Mobilome: prophages, transposons
*C*	90	102	84	109	Energy production and conversion
*G*	164	207	133	126	Carbohydrate transport and metabolism
*E*	94	110	95	124	Amino acid transport and metabolism
*F*	58	62	58	61	Nucleotide transport and metabolism
*H*	68	75	74	83	Coenzyme transport and metabolism
*I*	58	64	43	65	Lipid transport and metabolism
*P*	102	100	97	117	Inorganic ion transport and metabolism
*Q*	16	15	8	21	Secondary metabolites biosynthesis, transport and catabolism
*R*	151	158	122	168	General function prediction only
*S*	85	91	78	93	Unknown function
–	414	426	253	538	Not in cog

The 16S rRNA gene sequence obtained by sanger sequencing was identical to that obtained within the sequenced genome (Locus Taq : CJIAFGEE_00528, contic : FQRX01000011.1 , Per. Ident : 99.51% using Blast).

## Conclusion

On the basis of unique phenotypic features, including the MALDI-TOF spectrum, a 16S rRNA sequence similarity lower than < 98.65% and, an OrthoANI and a DDH values lower than 95% and 70% respectively with the phylogenetically closest species with standing in nomenclature, we formally propose strain Marseille-P2143^T^ as the type strain of *Anaerococcus urinimassiliensis* sp. nov., a new species within the genus *Anaerococcus*. *Anaerococcus urinimassiliensis* (u.ri.ni.mas.si.li.en’sis. N.L.adj.masc. urinimmassiliensis, composed of urini, from the latin urina, urine and massiliensis, from Massilia, the roman name of Marseille, France, where the strain Marseille-P2143 was first isolated. The colonies are thin, translucent and 50 µm in diameter. Cells are Gram-positive and anaerobic cocci. Cells have a diameter ranging from 0.5 to 0.7 µm. They do not produce catalase and oxidase, but exhibit alkaline phosphatase, leucine arylamidase, esterase lipase, α-galactosidase, naphthol-AS-BI-phosphohydrolase and β-galactosidase. d-glucose, d-galactose, d-maltose, d-lactose, d-fructose, N-acetyl-glucosamine, salicin, and sucrose are metabolized. The genome of strain Marseille-P2143 is 2,189,509 bp long with a 33.5 mol% G + C content. Its 16S rRNA gene sequence and whole-genome sequence are deposited in GenBank under accession numbers LN898272.1 and NZ_FQRX00000000.1, respectively. The type strain Marseille-P2143^T^ (= CSUR P2143 = DSM 103,473) was isolated from the urine of a 17-year-old boy suffering from autoimmune hepatitis and membranoproliferative glomerulo-nephritis. This new species implements the repertoire of human urinary tract known bacteria^44^.

### Description of *Anaerococcus urinimassiliensis* sp. nov

*Anaerococcus urinimassiliensis* (u.ri.ni.mas.si.li.en’sis. L. gen. masc. urini, of Urine and massiliensis referring to the Latin name of Marseille where strain Marseille-P2143 was cultivated). Cells are Gram-positive and non-motile, but they are negative for catalase and oxidase activities. They had a mean diameter of 0.6 µm. On blood agar after 48 h of incubation at 37 °C, colonies of strain Marseille-P2143 appear transluscent white. The optimum growth is observed at pH 7.5. Major cellular fatty acid was Hexadecanoic acid (57%), while unsaturated fatty acids such as 9-Octadecenoic acid and 9,12-Octadecadienoic were also detected with strain Marseille-P2143. The enzymes activities of alkaline phosphatase, esterase, esterase lipase, leucine arylamidase, cystine arylamidase, trypsin, naphthol-AS-BI-phosphohydrolase, α-galactosidase and β-galactosidase were positive using the API ZYM. Also, fructose, galactose, glucose, N-acetyl-glucosamine, salicin, maltose, lactose, and saccharose are fermented but not for mannose, methyl α-d-glucopyranoside, melibiose, turanose, erythritol, tagatose, adonitol, mannose, mannitol, arbutin, esculin, trehalose, sorbitol, inulin, melezitose and amygdalin. 16S rRNA and genome sequences of this new species are deposited in GenBank under accession numbers LN898272 and FQRX00000000, respectively. The genome size is 2.19 Mb with a G + C content at 33.47%. The type strain Marseille-P2143^T^ (= CSUR P2143 = DSM 103,473). The Marseille-P2143 strain was isolated in the urine of a young boy suffering from autoimmune hepatitis membranoproliferative glomerulonephritis.

### Deposit in culture collections

Strain Marseille-P2143^T^ was deposited in the French culture collection center, *Collection des Souches de l’Unité des Rickettsies* (CSUR), under the number CSUR P2143. And in the DSM collection under the number 103473.
